# Molecular epidemiology of helminth diseases of the humpback grouper, *Cromileptes altivelis*, as a pattern for mapping fish diseases in the Sunda Strait, Indonesia

**DOI:** 10.14202/vetworld.2021.1324-1329

**Published:** 2021-05-27

**Authors:** Sri Subekti, Muhammad Kholiqul Amiin, Hervina Benazir Ardiyanti, Muhammad Aiman Yudarana, Ivan Achmadi, Rizhar Eman Karunia Akbar

**Affiliations:** 1 Department of Marine, Faculty of Fisheries and Marine, Universitas Airlangga, Surabaya, Indonesia; 2 Department of Biotechnology Fisheries and Marine, Universitas Airlangga, Mulyorejo, Surabaya 60115, East Java, Indonesia

**Keywords:** humpback grouper, multiplex polymerize chain reaction, worm disease

## Abstract

**Background and Aim:**

*Neobenedenia* *girellae* and *Haliotrema* *epinepheli* are important but neglected helminth parasites of marine fish. The humpback grouper, the most relevant definitive host, harbors several *Neobenedenia* and *Haliotrema* spp. simultaneously on body surfaces and gills. These species can be distinguished morphologically This study aimed to identify *Neobenedenia* and *Haliotrema* spp. infestations in monogenean humpback grouper by multiplex polymerase chain reaction method, which seems to be widely distributed in the study area. Data can be used as a basis for mapping disease patterns in Strait waters.

**Materials and Methods:**

Eighty humpback groupers (*Cromileptes altivelis*) were collected from eight different areas in the Sunda Strait and examined using scrapings from body surfaces and gill lamellae followed by multiplex PCR for identification.

**Results:**

Parasites on body surfaces were recovered from 49 fish (61.2%) and were found on gill lamellae in 72 fish (90%) by microscopic examination. Endoparasites were absent. Ectoparasites identified included, *N. girellae*, *Neobenedenia melleni* eggs, *Neobenedenia pasifica*, *Neobenedenia longiprostata*, *Haliotrema eukurodai*, *H. kurodai*, *Haliotrema leporinus*, *Haliotrema dongshaense*, *Haliotrema angelopterum*, *Haliotrema aurigae*, *Haliotrema scyphovagina*, and *H. epinepheli*.

**Conclusion:**

The distribution of trematode disease in humpback grouper in Sunda Strait waters was revealed. All parasites were from genera, *Neobenedenia* and *Haliotrema*. Risks associated with these parasites should not be overlooked. Prevention and control programs need to be extended to other marine fish. Humpback grouper should be dewormed more frequently.

## Introduction

Humpback groupers are an important resource in Indonesian waters. This species has substantial marketing potential, especially for export. Humpback groupers were included in the 20 major Indonesian fish exports in 2017. Farmed fish are exported to many countries, including Japan, Taiwan, Malaysia, the United States, and several European nations [1].

However, parasites remain an unresolved problem for the successful cultivation of the species. Factors that may influence parasite incidence in marine aquaculture are density of fish, environmental conditions and water quality (temperature, salinity, and pH), fish handling, nutrition, feed resources, diet, and parasite/host relationships [2]. High stocking density encourages the spread of ectoparasites by direct transfer from fish to fish. Farmers who use trash fish caught locally as fodder for valuable species can transmit the parasite from the surrounding area to cultivated fish [3].

Epidemiological studies and molecular techniques can be used to control parasitic disease in humpback grouper. These methods assess incidence, distribution, and type of disease in a population. Epidemiological disease investigations begin when disease first arises. Subsequently, the study can focus on connecting site conditions, population risks, time, environmental characteristics, notable symptoms, and lesions (sores).

This study aimed to identify *Neobenedeni*a and *Haliotrem*a spp. infestations in monogenean humpback grouper by multiplex polymerase chain reaction method, which seems to be widely distributed in the study area. Data can be used as a basis for mapping disease patterns in Strait waters.

## Materials and Methods

### Ethical approval

Ethical approval was not required for this study; however, samples were collected as per standard sample collection procedure.

### Study period and locations

**Figure-1:**
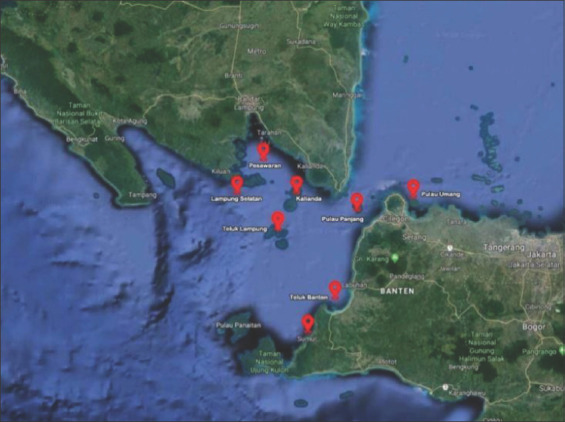
Map of Sunda Strait waters in Indonesia and its region, where humpback groupers samples were collected [Source: www.maps.google.com].

Sampling was conducted in May 2019, a total of 80 species of humpback grouper taken every day from eight locations floating net cages different in the waters of the Sunda Strait (four locations in Lampung Province and four sites in Banten Province): KJA A (KA) (5°31 ‘40.01 “LS105°14’ 54.60” E), KJA B (KB) (5°33 ‘43.36 “LS105°32’ 50.90” E), KJA C (KC) (5°44 ‘9:32’ LS105°35 ‘36.93 “E), KJA D (KD) (5°29’ 24.00” LS105°17 ‘12:24 “E), KJA E (KE) (6°38’ 33.59” LS105°34 ‘53.45 “E), KJA F (KF) (6°12’ 00:02” LS106°29 ‘43.40 “E), KJA G (KG) (5°55’ 36.36” LS106°09 ‘07.81 “E), and KJA H (KH) (6°16 ‘00.79 “LS106°28’ 5:19 ‘BT) (Figure-1).

### Fish collection

The number of fish samples (n) and biometrics are as follows: KJA A (KA) (146.2 g, 22.55 cm, n = 10); KJA B (KB) (127.8, 21.35 cm, n = 10); KJA C (KC) (89.5 g, 19.05 cm, n = 10). KJA D (KD) (110.2 g, 20.8 cm, n = 10); KJA E (KE) (193.9 g, 23.85 cm, n = 10); KJA F (KF) (160.4 g, 22.25 cm, n = 10); KJA G (KG) (166.5 g, 21.86 cm, n = 10); and KJA H (KH) (144.8 g, 21.2 cm, n = 10). Fish samples were taken to the Laboratory of the Fish Health and Environment Marine Culture Centre in Lampung Province, Indonesia, for examination and sampling of parasitic worms.

### DNA extraction and multiplex polymerase chain reaction (PCR)

RNA extraction used TriSol solution (Kit IQ2000TM). Up to two pieces of scales, fins, or gills (300 mg), were place in 1 mL of PBS (1 mL) and tissues disrupted with a tissue disruptor until well blended. Two hundred and fifty microliters of sample and 750 μL of TriSol were mixed and centrifuged at 12,000 g for 5 min at 4^o^C. The supernatant was then incubated for 5 min. One hundred and fifty microliters chloroform was added, incubation continued for 2-3 min, and mixtures centrifuged at 12,000 g for 5 min at 4^o^C. Three phases were obtained; RNA-se, interphase, and red phenol chloroform. The RNA phase was mixed with 225 mL of 100% ethanol, by inverting and incubated for 2-3 min. The mixture was then centrifuged at 2000 g for 5 min at 4°C. As much as, 200 μL supernatant was then placed into a new 1.5 mL microtube and isopropanol. The mixture was incubated for 30 min with inverting and the mixture was then centrifuged using back vortex at 2000 g for 5 min at 4°C. The supernatant was then discarded and the process repeated 3 times. The final pellet was suspended in as much as 1.5 mL 70% ethanol, then centrifuged again at 2000 g for 5 min at 4°C. The supernatant was removed and the pellet dried in the air for 5-10 min. The dried pellet was dissolved in 200 mL of 8 mM NaOH. Pellet RNA was then ready for use or storage after centrifuging again at 12,000 g for 10 min at 4°C. The supernatant was used to develop the whole DNA product.

DNA extraction used a Qiagen DNA Mini Kit (Germany) with the protocol for tissue. Extraction followed the manufacturer’s instructions with some modifications. Primers used for multiplex PCR are shown in Table-1. Amplification reactions used a 50 μL volume, containing 28 μL Master Mix, 5 μL primers, 1 μL MM, 5 μL 10× buff, 5 μL dNTP, 2 μL MgSO_4_, 2 μL Primer F, 2 μL Primer R, 1 μL KOD ver. 2, and 5 μL template DNA. PCR cycling conditions used one cycle of 2 min at 95°C for initial denaturation, 2 min primary denaturation at 93°C; followed by 35 cycles of denaturing at 93°C for 1 min, annealing at 51°C for 45 s, and extension at 72°C for 2 min and a final extension at 72°C for 5 min. Five microliters of each PCR product were analyzed by electrophoresis on 2% agarose gels stained with Safe Stain. A 1000 bp molecular size ladder was run together with samples to determine fragment lengths. PCR products were visualized under ultraviolet light (UVI doc, England).

**Table-1:** Primers, targets, and sequences applied for multiplex PCR.

Target species	Target gene	Primer designation	Sequences (5’-3’)	Amplicon length (bp)
*Neobenedenia girellae*	rRNA	ITS 1	GATAGGCCCGTTCAGGTTGG	768 bp
		ITS 2	AGGCGTGGCTTCTTGACAAA	
*Haliotrema epinepheli*	rRNA	ITS 1	CGCTTCAAGTCGTTCTGGGA	821 bp
		ITS 2	CCAGCGGATCGAAATCAACG	

PCR=Polymerase chain reaction

## Results

**Figure-2:**
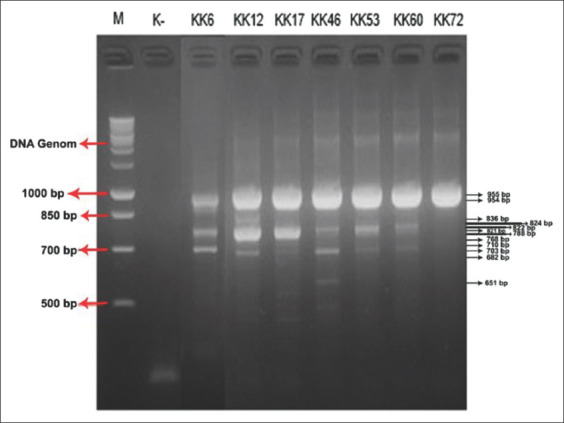
The results of PCR analysis on the scales of humpback grouper fish (*Cromileptes altivelis*). M is the 1000 bp ladder. K- is negative control. Lanes KK 6, 12, 17, 46, 53, 60, and 72 are 650-955 bp PCR product representative of genera *Neobenedenia*.

Forty-nine of 80 examined fish (61.2%) showed at least one parasite on scales and 72 of 80 fish (90%) showed at least one parasite on gills. *Neobenedenia girellae* and *Haliotrema epinepheli* were observed in 80 samples. Parasites were used for multiplex PCR analysis. Amplification of 954, 824, and 710 bp fragments of target rRNA was observed in the KK 6 sample. The KK 12 sample displayed amplification of 955, 768, and 682 bp fragments; KK 17 exhibited amplification of 955 and 788 bp fragments. The KK 46 sample displayed more DNA bands than other samples, with amplification of 955, 822, 703, and 651 bp fragments. The KK 53 sample showed amplification of 955 and 821 bp fragments: KK 60 exhibited amplification of 954 and 836 bp fragments. Only the 954 bp fragment was amplified from KK 72. All samples indicated infestation with *Neobenedenia* spp. (Figure-2).

**Figure-3:**
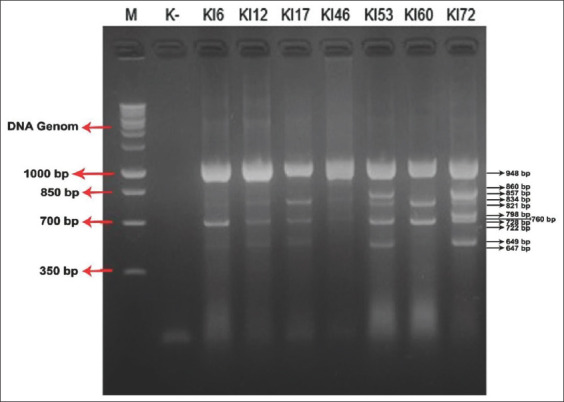
Gel electrophoresis of the polymerize chain reaction (PCR) products gills of humpback grouper fish (*Cromileptes altivelis*). M is the 1000 bp ladder. K- is negative control. Lanes KI 6, 12, 17, 46, 53, 60, and 72 are 647-948 bp PCR product representative of genera *Haliotrema*.

Positive results were also observed in gill samples. The KI 6 sample displayed 948 and 722 bp fragments; KI 12-948 and 728 bp fragments; KI 17-948, 834, and 728 bp fragments; and KI 46-948 and 760 bp fragments. The KI 53 sample showed amplification of 948, 857, 728, and 647 bp fragments, the most fragments among gill samples. The KI 60 sample exhibited amplification of 948, 821, and 722 bp fragments and KI 72-948, 860, 798, and 649 bp fragments. Seven samples (KI 6, 12, 17, 46, 53, 60, and 72) displayed the 948 bp and three samples (KI 12, 17, and 53) showed the 728 bp fragments. All samples indicated infestation with *Haliotrema* spp. (Figure-3).

## Discussion

A relatively high rate of parasitic infestation on scales and gills of *C*. *altivelis* was found in the study region of Sunda Strait, Indonesia. Several parasite species of monogenean trematode were identified, all in genera *Neobenedenia* and *Haliotrema*. Monogenean trematode parasites of humpback grouper are important because they decrease fish endurance that leads to further infestation or secondary infection by bacteria and viruses [4]. Parasitic worms infest fish through water that has been contaminated by affected fish in aquaculture and through direct contact with other fish infected by the worms. The spread of unhealthy fish has significant consequences, especially when stocking density is too high [5].

Data on the prevalence of parasitic infestation on fish scales can be used to inform control measures to minimize the risk of disease transmission among fish. The overall prevalence of parasites on scales and gills was 62.1% and 90%, respectively, among 80 fish analyzed. This infection rate is greater than reported in waters of Chile (24%) [6] and Mexico (53%) [7], but less than in Japan (98.7%) [8]. The rate of parasitic infestation in gills is greater than reported in Vietnam (2.2%) [9] and Japan (62%) [10], but less than Mexico (100%) [11]. Among parasites detected, the trematode family, Capsilidae, contains two closely related genera, *Neobenedenia* and *Benedenia*. Species in these genera show different levels of pathogenicity that makes them the subjects of intensive epidemiological study [12].

Further, one parasite of the family Diplectanidae in the genus *Haliotrema* is of particular concern in epidemiological studies [12]. Accurate diagnoses of monogenean parasites provide key information for controlling infection of marine fish. Examination of scales and gills is not sufficiently accurate for epidemiological purposes because *Neobenedenia* and *Benedenia* spp. are similar, as are *Haliotrema* and *Diplectanum* spp. Likewise, immunological methods for copro-antigen detection and serum antibodies are not sufficiently specific to differentiate among pathogens at a species level [13]. To overcome these limitations, various molecular approaches, such as high-sensitivity multiplex PCR, have been developed [13,14].

Use of multiplex PCR in our study showed 61.2% infestation rate with *N. girellae* and 90% with *H. epinepheli* of grouper from Sunda Strait waters. In regions outside Indonesia, different infestation rates with these species were reported in studies the multiplex PCR method. Kurniawan [15] and Subekti *et al*. [16] reported the rate of infestations of *N. girellae* as 44.44% and 56.67%, respectively. The same researcher also reported an attack rate of *H. epinepheli*, respectively, at 27.78% and 26.67%. Several environmental and socioecological factors are involved in the dispersal of *N. girellae* and *H. epinepheli* among hosts. These factors control the prevalence of infestations. The incidence rate observed in fish may reflect a lack of control over fish and water quality, as well as low parasite levels; however, prevention of proliferation of monogenean parasites is still needed in the Sunda Strait. Trematode parasites develop rapidly. Eggs that hatch in water swim using cilia, called onchomiracidium, to find suitable hosts [2].

DNA bands amplified from rRNA from study fish display different amplicon lengths. Seven samples of scales exhibit amplification of 954-955 bp fragments. Parasites with 954-955 bp amplicon lengths can be classified as *Neobenedenia melleni* eggs [17]. *N. melleni* worm larvae show amplicon lengths of 651 and 703 bp [17]. The KK 46 sample displayed such amplicon lengths of 651 and 703 bp. Adult *N. melleni* worms appeared in the KK 46 samples with an amplicon length of 822 bp (Figure-3). These results are also consistent with previous research [17].

Other scale samples also showed different amplicon lengths. These results are consistent with Brazenor *et al*. [13], where *N. girellae* were associated with amplicon lengths of 682 bp (KK 12), 710 bp (KK 6), 768 bp (KK 12), 824 bp (KK 6), and 836 bp (KK 60) (Figure-2). In contrast, samples showing an amplicon length of 788 bp (KK 17) (Figure-2) are classified as *Neobenedenia pasifica*. Other studies also found *Neobenedenia longiprostata* with an amplicon length of 821 bp (KK 53) [12]. Some DNA bands found suggest a parasite dispersal pattern that involves the surface of a humpback grouper bodies. Multiplex PCR results show that *N. girellae*. *N. melleni*, *N. pasifica*, and *N. longiprostata* were also identified.

Multiplex PCR of gill specimens successfully amplified DNA, with bands indicating amplicon lengths of 948 bp (KI 6, KI 12, KI 17, KI 46, KI 53, KI 60, and KI 72). This finding is consistent with a previous report [18] of amplicon lengths of *Haliotrema* spp. of 948-1000 bp. Further, *Haliotrema* spp. also display amplicon lengths of 720-730 bp [19], consistent with current results showing amplicon lengths of 722 bp (KI 6 and KI 60) and 728 bp (KI 12, KI 17, and KI 53). Further, several other DNA bands with different amplicon lengths were observed in samples KI 17 (834 bp), KI 46 (760 bp), KI 53 (647 and 857 bp), KI 60 (821 bp), and KI 72 (649, 798, and 860 bp) (Figure-3). Several parasitic species infest gills of humpback grouper in the waters of the Sunda Strait.

The targets of the current study were specimens of *H. epinepheli*. These worms were found and characterized by DNA bands with an amplicon length of 821 bp in the KI 60 sample. Similar investigation reported amplicon lengths of 800-821 bp in *H. epinepheli* [14]. Further, Sun *et al*. [14] also reported *Haliotrema eukurodai*, *H. kurodai*, *Haliotrema leporinus*, and *Haliotrema dongshaense*, displaying amplicon lengths of 834 bp (KI 17), 760 bp. (KI 46), 857 bp (KI 53), and 860 bp (KI 72), respectively. Multiplex PCR on seven samples of gill humpback grouper indicated only species of genus *Haliotrema*. *H. epinepheli*, which was targeted in this study, was identified.

## Conclusion

Parasitic infestation by genera *Neobenedenia* and *Haliotrema* is endemic in humpback grouper in the Sunda Strait, Indonesia. The presence of ectoparasite infestations occurs because of poor fish management and uncontrolled water quality that fluctuates seasonally. The pattern of worm disease mapping in Sunda Strait waters is horizontal through contact with aquaculture media, such as floating net cages that have been contaminated by ectoparasitic worm larvae or by direct contact with other fish that are infected with ectoparasites. Differences in infestation rate within Sunda Strait waters depend on factors, such as definitive hosts. Additional study is required to determine the prevalence of parasites on all hosts in the study areas.

## Authors’ Contributions

MHK: Provided the research proposal and performed laboratory works. SS: Supervised the study and revised the manuscript. HBA: Prepared the study design. MAY, IA, and REKA: Collected the samples and managed the laboratory for the project. MKA: Drafted the manuscript. All authors read and approved the final manuscript.
